# Phytochemical Screening and Biological Studies of Leaf Extract of *Ipomoea Batatas* (L.) Lam

**DOI:** 10.1155/tswj/6660150

**Published:** 2026-06-22

**Authors:** Rabindra Prasad Timilsina, Susmita Bhandari, Bechan Raut

**Affiliations:** ^1^ Department of Pharmacy, MMIHS, Tribhuvan University, Kathmandu, Nepal, tribhuvan-university.edu.np; ^2^ Department of Pharmacy, Suvekchya Polytechnic College, Kathmandu, Nepal

**Keywords:** antimicrobial activity, antioxidant capacity, GC-MS, *Ipomoea batatas* (L.) Lam., sweet potato, total flavonoid content, total phenolic content

## Abstract

Medicinal plants have been utilized in indigenous healthcare systems for the therapeutic management of various diseases. A significant proportion of modern therapeutic agents originate from plant sources, and Nepal is reported to possess more than 900 species of medicinal plants with potential medicinal value. *Ipomoea batatas* (L.) Lam., widely known as a sweet potato, is a herbaceous vine widely recognized in Nepalese traditional medicine. The plant has been associated with several bioactivities, like antifertility, anti‐inflammatory, antimutagenic, antibacterial, and antidiabetic effects. This study examined the phytochemical constituents, antioxidant potential, and the antimicrobial activity of crude hydroethanolic leaf extract of *I. batatas* (L.) Lam. Preliminary screening of phytoconstituents revealed the detection of glycosides, saponins, flavonoids, tannins, and terpenoids. Gas chromatography–mass spectrometry (GC‐MS) analysis identified four compounds: phytol, caffeic acid, peonidin, and 2‐phenyl dodecane. The total phenolic content was determined to be 123.938 mg GAE/g, whereas total flavonoid content was determined to be 152 mg QE/g. The reducing power assay demonstrated a concentration‐dependent increase in absorbance values (0.164–0.471) at extract concentrations of 10–50 *μ*g/mL, indicating enhanced antioxidant activity. The IC_50_ value obtained from the DPPH radical scavenging assay was 15 *μ*g/mL. However, the extract did not exhibit antimicrobial activity against *E. coli* or *S. aureus*. These findings suggest that the extract of *I. batatas* is rich in bioactive compounds and may represent a promising source of natural antioxidant agents.

## 1. Introduction

Medicinal plants have made a significant contribution to the prophylaxis and treatment of disease since early human civilization and continue to be a significant source of pharmacologically active compounds in modern medicine [1]. A substantial proportion of currently used pharmaceutical products are derived from plants or are based on phytochemicals originally identified from botanical sources. Due to their wide availability, affordability, and cultural acceptance, herbal medicines remain extensively utilized, particularly in developing countries, and are generally considered effective for the treatment of various diseases and disorders [2].

**Figure 1:**
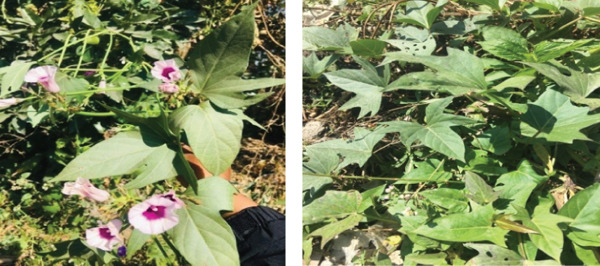
*Ipomoea batatas* (L.) Lam. with its flower; Photograph taken by authors at sample collection site.

*Ipomoea batatas* (L.) Lam., popularly referred to as sweet potato, is a dicotyledonous member of the family *Convolvulaceae* [3] (Figure 1). It is cultivated worldwide and serves as an important food crop owing to its high nutritional value. Sweet potato leaves are recognized as a significant reservoir of nutrients and essential minerals including manganese, copper, potassium, and iron [4]. In Nepal, the plant also holds cultural and religious significance and is traditionally harvested and consumed during festivals such as Thula Ekadashi and Makar Sankranti [5].

Phytochemical investigations have revealed that sweet potato leaves contain diverse secondary metabolites, comprising alkaloids, tannins, glycosides, saponins, terpenoids, flavonoids, coumarins, and phenolic acids. These biologically active constituents have demonstrated a broad range of pharmacological activities [6]. Ethnopharmacological studies further indicate that *I. batatas* leaves collected from regions such as Malaysia and Brazil have demonstrated strong antioxidant potential and have historically been employed in the management of oral diseases, largely attributed to their high content of flavonoids, anthocyanins, *β*‐carotene, and phenolic compounds [7, 8].

Oxidative stress, resulting from excessive production of free radicals, is strongly associated with the pathogenesis of numerous chronic disorders. Although synthetic antioxidants are widely used to counter oxidative damage, their prolonged consumption has been associated with potential adverse health effects, comprising an increased chance of cancer and liver toxicity [9, 10]. Consequently, there is a growing scientific interest in identifying natural antioxidants that are safe, effective, and economically viable. *I. batatas* possesses considerable medicinal significance, and different plant parts are employed in ethnomedical practices. In particular, *I. batatas leaves* have been used for the management of Type 2 diabetes mellitus, inflammatory disorders, and oral diseases in regions such as Ghana and Brazil. Additionally, sweet potato leaves have traditionally been consumed to alleviate conditions including anemia, hypertension, and diabetes, whereas other parts of the plant have been utilized in the treatment of prostatitis [5]. Elevated levels of polyphenols and vitamins found in the leaves contribute to their free radical scavenging ability and associated antibacterial, anti‐inflammatory, and antihypertensive activities [11].

In view of the pharmacological importance of *I. batatas* and the limited scientific data on Nepalese plant material, the current study was undertaken to assess the phytochemical constituents, antioxidant activity, and antimicrobial potential of the crude hydroethanolic leaf extract of *I. batatas* (L.) Lam. The findings of the present study may provide scientific support for the traditional medicinal use of the plant and highlight its potential for future pharmacological applications.

## 2. Materials and Methods

### 2.1. Plant Sample Collection

Fresh leaves of flesh‐white *I. batatas* (sweet potato) were collected in November from Budhanilkantha‐11, Kathmandu district, Nepal. The plant material was identified based on its taxonomic characteristics and properly authenticated at the National Herbarium and Plant Laboratories (NHPL), Godawari‐03, Lalitpur, Nepal.

### 2.2. Bacterial Sample Collection

Bacterial strains susceptible to standard antibiotics were selected for antimicrobial evaluation. Standard control strains *Staphylococcus aureus* ATCC 25923 and *Escherichia coli* ATCC 25922 were procured as pure culture from the Natural Product Research Laboratory, Thapathali, Kathmandu.

### 2.3. Positive Control

Commercially available gentamicin and doxycycline were used as positive control antibiotics during antibacterial assays. Gentamicin was used against *E. coli*, whereas doxycycline was employed for *S. aureus*.

### 2.4. Preparation of Plant Extract of Crude Leaves [8]

Fresh and mature leaves of *I. batatas* (L.) Lam. were carefully washed with tap water to eliminate surface impurities and subsequently shade‐dried for 2 weeks at ambient temperature. The dried leaf samples were then powdered using an electric blender and stored in tightly sealed airtight glass containers.

**Figure 2:**
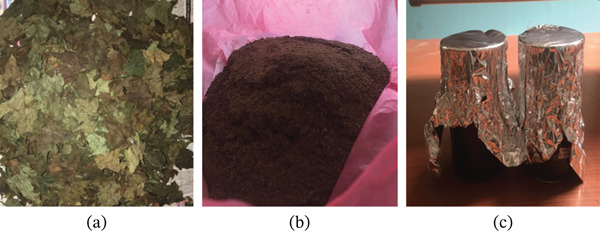
*Ipomoea batatas* (L.) Lam. leaves sample processing. (a) Shade‐dried leaves, (b) powder form of leaves, and (c) maceration extraction.

Plant material was extracted using the maceration method. Approximately 200 g of plant powdered was submerged in 1000 mL of 70% (v/v) ethanol and kept at ambient temperature for 7 days with occasional stirring under aseptic conditions. The resulting mixture was first filtered through muslin cloth and then filtration using Whatman Grade 1 filter paper. The filtrate underwent vacuum concentration at 40°C using a rotary evaporator set at 100 rpm until dryness was achieved. The crude extract was preserved at 4°C in light‐ and moisture‐protected conditions until further analysis (Figure 2).

The percentage yield was computed using the following formula:

Percentage extractive yield=Weight of sampleg/Weight of extract g×100.

### 2.5. Preliminary Phytochemical Screening [12, 13]

Qualitative phytochemical screening of the hydroethanolic extract of the leaves of *I. batatas* (L.) Lam. was performed using established laboratory procedures. The extract was examined for the occurrence of major secondary plant constituents, such as glycosides, alkaloids, proteins, carbohydrates, flavonoids, saponins, phenols, terpenoids, steroids, anthraquinones, and tannins. The presence or lack of these constituents was evaulated based on their characteristic color changes observed during the respective qualitative reactions [12, 13].

### 2.6. Gas Chromatography–Mass Spectrometry (GC‐MS) Profiling of Hydroethanolic Lleaf Eextract

GC‐MS analysis was performed to identify the bioactive constituents within the hydroethanolic extract of *I. batatas* leaves. The characterization was conducted at the Nepal Academy of Science and Technology (NAST), Khumaltar, Lalitpur, Nepal.

GC‐MS analysis was performed with a Shimadzu GC‐MS QP2010 system (Kyoto, Japan) equipped with an RTx‐5MS fused silica capillary column (30 m × 0.25 mm × 0.25 *μ*m). Helium (> 99.99% purity) was served as the carrier gas at a linear velocity of 36.2 cm/s. The system was operated maintaining a total flow rate of 3.9 mL/min, column flow of 0.95 mL/min, and purge flow rate of 3.0 mL/min. A sample volume of 1 *μ*L was injected in split‐less mode at an injector temperature set to 280°C.

The oven temperature program began at 100°C and was raised to 250°C at a heating rate of 15°C/min with a holding time of 1 min, then ramped to 280°C at a rate of 30°C/min (1 min hold), and finally raised up to 300°C at a heating rate of 15°C/min with a final holding time of 11 min. The ion source temperatures was set at 200°C and interface temperatures at 280°C with a solvent cut time of 3.5 min. Mass spectra were recorded over a range of 40–500 m/z with a total analysis time of 20 min. Identification of the compounds was performed by comparison of the obtained mass spectra against the NIST08 mass spectral library.

### 2.7. Determination of Total Phenolic Content (TPC) [14, 15]

The Folin–Ciocalteu colorimetric method was used to determine the TPC of the hydroethanolic leaf extract. Briefly, 1 mL of the extract prepration (1 mg/mL) was combined with 5 mL of Folin–Ciocalteu reagent (1:10, v/v diluted with distilled water), followed by 4 mL of 7% (w/v) sodium carbonate solution. The reaction mixture was incubated at 40°C for 30 min, and the absorbance was subsequently recorded at 760 nm using a UV–Visible spectrophotometer.

A calibration curve was constructed using standard solutions of gallic acid (10–200 *μ*g/mL), and TPC was reported as milligram of gallic acid equivalents per gram of dry extract (mg GAE/g).

### 2.8. Spectrophotometric Determination of Total Flavonoid Content (TFC) [15, 16]

The aluminum chloride colorimetric assay was employed to determine the TFC of the hydroethanolic leaf extract. Quantification was performed using a calibration curve prepared with quercetin standards (10–200 *μ*g/mL). Total amount of flavonoid was expressed as milligram of quercetin equivalents per gram of extract (mg QE/g) on a dry weight basis.

### 2.9. Evaluation of Antioxidant Capacity

#### 2.9.1. Assessment of Reducing Power Activity (RPA): [17, 18]

The reducing power of the hydroethanolic extract was assessed using the ferric reducing power assay. Absorbance was measured at 700 nm using a UV–Visible spectrophotometer. Ascorbic acid was used as the standard, and increased absorbance indicated higher reducing power of the sample.

#### 2.9.2. DPPH Free Radical Scavenging Activity [10]

The antioxidant activity of the hydroethanolic leaf extract was assessed using the DPPH (2, 2‐diphenyl‐1‐picryl hydrazyl) free radical scavenging method [10]. The absorbance was measured at 517 nm using a UV–Visible spectrophotometer, with methanol containing DPPH used as a blank. The percentage inhibition was calculated, and the IC_50_ value was determined using a regression equation.

### 2.10. Determination of Anti‐Bacterial Activity [19]

The antibacterial activity of the extract was determined using the agar well diffusion method on Mueller–Hinton Agar (MHA) as the culture medium. A standardized bacterial suspension was evenly distributed over the dried surface of MHA plates with sterile cotton swabs to obtain a confluent lawn culture. Using a sterile cork borer, four wells each of 6 mm diameter were aseptically bored into the inoculated agar medium. The wells were filled with varying concentrations of the sample extract (12.5, 25, 50, and 100 mg/mL). Gentamicin (1 mg/mL) against *E. coli* and doxycycline (0.2 mg/mL) against *S. aureus* was employed as positive controls, whereas 1% dimethyl sulfoxide (DMSO) served as negative control. Control plates were prepared under identical experimental conditions.

Following incubation at 37°C for 24 h, antibacterial activity was determined by measuring the diameter of the zones of inhibition (ZOI) in millimeters using a digital Vernier caliper. The efficacy of the extract was evaulated by comparing the inhibition zones with those produced by the standard antibiotics. A larger inhibition zone indicated stronger antimicrobial potential of the extract against the microorganisms tested.

## 3. Result and Discussion

### 3.1. Extractive Value

The dried extract obtained after maceration of *I. batatas* (L.) Lam. leaves was accurately weighed, and the yield percentage was calculated. The extractive value was determined to be 7.73% (Table 1). This yield is lower than that reported in a previous study [4], which documented a 34.41% extractive value for the crude leaf extract. Likewise, research conducted in Indonesia [7] reported a 19.821% yield from leaves extracted by maceration using 96% ethanol. Variations in extractive yield may be due to factors including geographical source of the plant material, harvesting time, solvent composition and its concentration, extraction technique, and laboratory conditions during processing.

**Table 1:** Extractive value of *Ipomoea batatas* (L). Lam. leaves.

Plant	Solvent	Extraction time	Percentage extractive value
*Ipomoea batatas* (L). Lam. leaves	70% ethanol	7 days	7.73%

### 3.2. Phytochemical Screening

Preliminary qualitative analysis of the extract revealed the occurrence as well as absence of distinct classes of phytoconstituents. The investigated different secondary metabolites included tannins, alkaloids, saponins, flavonoids, glycosides, terpenoids, proteins, carbohydrates, and steroids (Table 2).

**Table 2:** Phytochemical screening result of *Ipomoea batatas* (L.) Lam. extracts ([*+*] present, [−] absent).

S.N.	Secondary metabolites	Name of test	Result
1	Tannins	FeCl_3_ 0.1%	**+**

2	Alkaloids	Mayer′s test	**+**
		Dragendroffs test	**+**
		Hager′s test	**+**
		Wagner′s test	**+**
3	Saponins	Foaming test	**+**

4	Flavonoids	Shinoda test	**+**
		Alkaline reagent test	**+**
		Zn‐hydrochloride test	**−**

5	Glycosides	Reducing sugar	**+**
		Anthraquinones glycoside	**−**
		Cardiac glycoside	**−**

6	Terpenoids	Salkowski′s test	**+**
7	Protein	Biuret test	**−**
8	Carbohydrate	Molish′s test	**−**
9	Steroids	Salkowski test	**−**

The observations obtained in this investigation are corroborate with the previous findings reported by Mayasari et al. [7] and comparable with earlier studies carried out by Osuntokun et al. [6] and Pochapski et al. [8]. Those studies also documented the occurrence of alkaloids, glycosides, flavonoids, saponins, tannins, terpenoids, steroids, and cardiac glycosides in hydroethanolic extracts. Differences in the detection of certain constituents, including alkaloids, steroids, and cardiac glycosides, may be ascribed to variations in geographical source, harvesting period, ambient conditions, and extraction parameters.

Alkaloids are recognized for exhibiting diverse pharmacological activities such as antiviral, antispasmodic, antiparkinsonian, antihypertensive, antitussive, and analgesic effects [20]. Flavonoids are reported to exhibit antioxidant, antibacterial, antiviral, anti‐inflammatory, and antithrombotic properties, and contribute to the reduction of oxidative stress associated with cardiovascular disorders [21]. Terpenoids demonstrate multiple bioactivities including antiallergic, antioxidant, antiviral, antiangiogenic, antiallergic, anticancer, and spasmolytic effects [22]. Tannins are known for their antioxidant, anticarcinogenic, antimutagenic, and antimicrobial potential [23]. Saponins, composed of an aglycone moiety linked to carbohydrate chains, demonstrate diverse pharmacological activities including antiallergic, antiviral, anti‐inflammatory, antimicrobial, anticancer, antioxidant, and immunomodulatory actions [24].

### 3.3. GC‐MS Analysis

**Figure 3:**
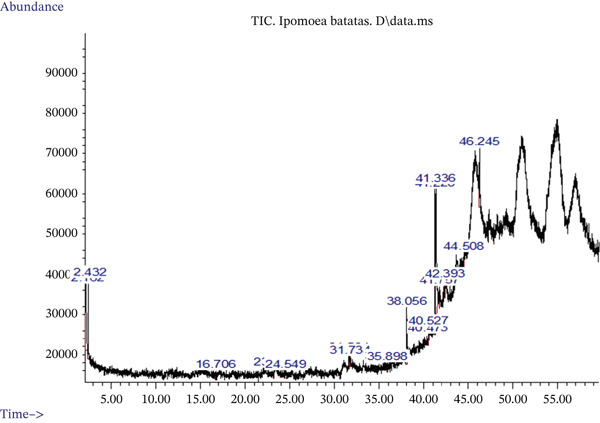
Chromatogram under GC‐MS analysis of the extract.

**Figure 4:**
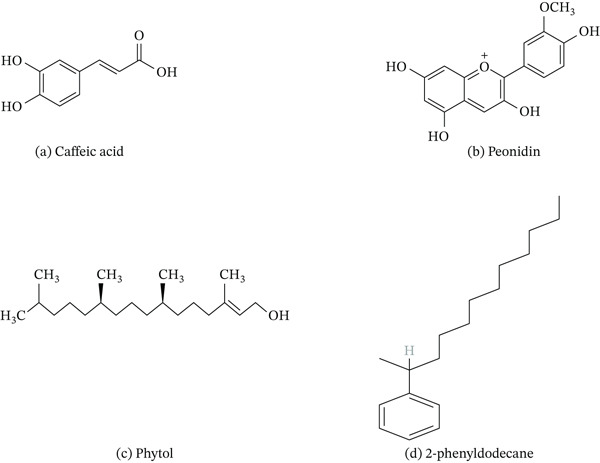
Chemical structure of identified compounds through GC‐MS analysis: (a) Caffeic acid, (b) peonidin, (c) phytol, and (d) 2‐phenyldodecane.

GC‐MS profiling of the *I. batatas* (L.) Lam. hydroethanolic leaf extract resulted in the identification of four predominant phytoconstituents with documented pharmacological significance (Table 3). The corresponding chromatographic pattern is presented in Figure 3, whereas Figure 4 illustrates the chemical structures of the reported compounds.

**Table 3:** GC‐MS identified compounds.

No.	Name of compound	Molecular formula	Molecular weight	Library similarity (NIST08, %)	Peak area (%)	Reported activity
1	Caffeic acid	C_9_ H_8_O_4_	180.16	91.4	18.6	Antioxidant, Anti‐inflammatory, Anticarcinogenic, Antidiabetic
2	Phytol	C_20_H_40_O	296.5	95.8	34.2	Antioxidant, Antinociceptive, Anti‐inflammatory, Antiallergic
3	Peonidin	C_16_H_13_O_6_^+^	301.27	89.7	21.9	Antioxidant, Anticarcinogenic
4	2‐phenyldodecae	C_18_H_30_	246.4	87.2	12.4	Antioxidant

As reported by Nguyen et al. [25], several phytochemicals were found in sweet potato leaves, including caffeic and caffeoylquinic acid derivatives, chlorogenic acid, quinic acid, anthocyanins, quercetin, myricetin, fisetin, morin, a 16‐amino‐acid peptide, and omega‐3 fatty acids. Among these, caffeic acid and peonidin were also identifed in the present study. In contrast, Kurniasih and Saputri [26] identified 1, 4‐benzenediol, hydroquinone, benzenesulfonic acid (reported to possess antibacterial activity), and hexadecanoic acid in the ethanol fraction of purple sweet potato leaves collected in Indonesia. Similarly, research from Nigeria [27] documented *n*‐hexadecanoic acid, cyclotrisiloxane (hexamethyl), and 16‐pregnenolone as the major bioactive constituents in GC‐MS analysis.

Differences in the phytochemical profile reported across studies can be ascribed to variability in geographic origin, climatic conditions, extraction procedures, solvent systems, and reagent concentrations employed during analysis.

### 3.4. Quantitative Screening

#### 3.4.1. TPC

**Figure 5:**
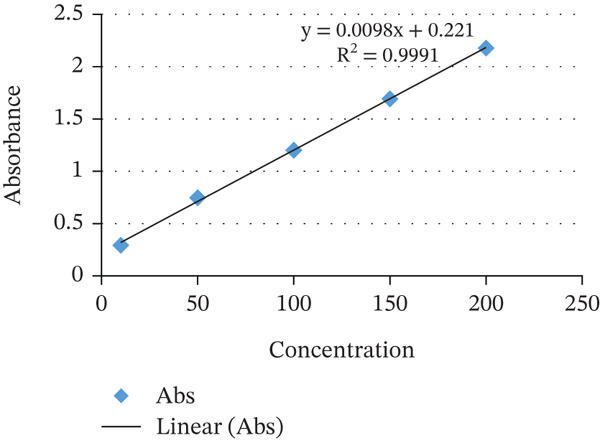
Calibration curve of standard gallic acid.

The absorbance readings of various concentrations of gallic acid standard, along with the plant extract, were recorded to construct a calibration curve (Table 4 and Figure 5).

**Table 4:** Absorbance value of gallic acid measured for the calibration curve.

Concentration(*μ*g/ml)	Absorbance at 760 nm
10	0.292
50	0.747
100	1.201
150	1.692
200	2.177
Extract	1.4356

From the curve obtained, we have

Y=0.00980.221+

R2=0.9991

Using the calibration equation derived from the standard curve, the TPC of *I. batatas* leaf extract was calculated as 123.938 mg GAE/g. This value exceeds than that reported in a previous study [4], which documented 112.98 ± 4.14 mg GAE/g. The observed variation may be attributed to differences in extraction techniques.

A comparative assessment of TPC among different cultivars of *I. batatas* leaves in the United States [28] indicated that ′O′Henry′ exhibited the greatest phenolic content (10.50 ± 1.04 mg), whereas ′Centennial′ recorded the lowest value (7.29 ± 0.62 mg). Both values are considerably lower than the result obtained in the present investigation. The study suggested that varietal characteristics play a major role in influencing phenolic levels when environmental and cultivation conditions are kept constant. Additionally, findings reported by Ishiguro [29] and Islam et al. [30] support that *I. batatas* leaves possess greater phenolic concentrations compared to other plant parts, including storage roots and potato tubers.

#### 3.4.2. TFC

**Figure 6:**
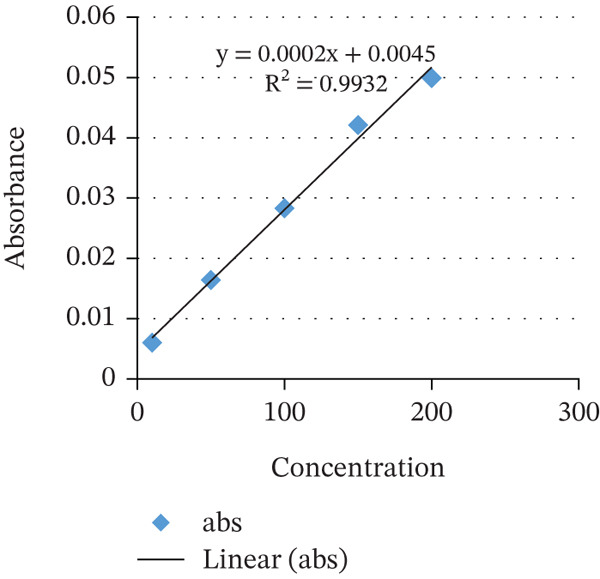
Calibration curve of standard quercetin.

The absorbance readings of quercetin standard solutions at different concentrations, along with the plant extract, were recorded to generate a calibration curve (Table 5 and Figure 6).

**Table 5:** Absorbance value of quercetin for calibration curve.

Concentration(*μ*g/ml)	Absorbance at 510 nm
10	0.006
50	0.0164
100	0.0283
150	0.0421
200	0.0499
Extract	0.0349

The linear regression equation derived from the reference curve was:

Y=0.00020.0045x+

R2=0.9932

Using this equation, the TFC of *I. batatas* leaf extract yielded 152 mg QE/g. This value is greater than that reported by Zang et al. [4], which documented 56.87 ± 5.69 mg QE/g. The difference may be attributed to the ultrasound‐assisted extraction technique performed at 50°C in their study. A comparative study conducted in the United States [28] evaluating different varieties of *I. batatas* leaves showed that ′O′Henry′ contained the highest flavonoid content (4.26 mg), whereas ′Centennial′ exhibited the lowest TFC (2.30 mg). Both values are substantially lower than the result obtained in the present investigation.

Previous studies have demonstrated a strong correlation between antioxidant capacity and the phenolic and flavonoid composition of plant materials as presented by Gong et al. [31]. The flavonoid content determined in the present study exceeds the values documented by Ghasemzadeh et al. [32]. However, direct comparison between studies is limited due to differences in solvent systems, extraction procedures, and analytical methodologies. Zhang et al. [4] reported total phenolic, total flavonoid, and polysaccharide contents in leaves as 112.98 mg GAE/g, 56.87 mg RE/g, and 49.41 mg GE/g, respectively, which were considerably greater than those in petioles. Similarly, a study from China [33] reported total phenolic as contents 135.9 mg GAE/g and TFCs as 5.8 mg RE/g, with ethanol used as the extraction solvent. Higher phenolic and flavonoid concentrations are generally associated with enhanced antioxidant capacity.

### 3.5. Antioxidant Activity

#### 3.5.1. Determination of Reducing Power

**Figure 7:**
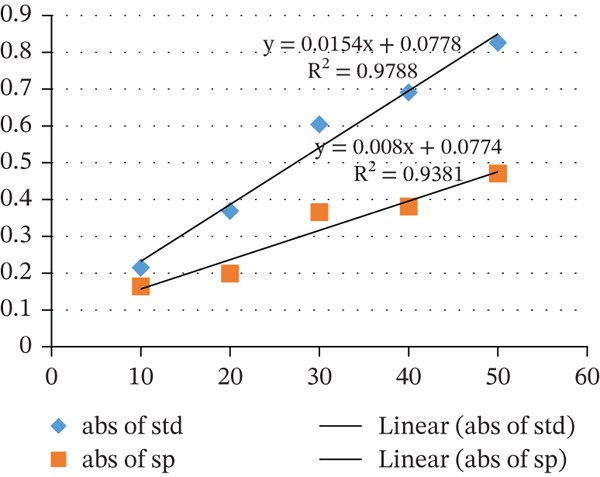
Determination of reducing power of hydroethanolic extract and standard ascorbic acid.

The absorbance values of ascorbic acid (standard) and the hydroethanolic leaf extract (test sample) at different concentrations were recorded at 700 nm (Table 6 and Figure 7). An increase in absorbance reflects enhanced reducing capability, which corresponds to greater antioxidant potential. Both the standard and the extract exhibited a concentration‐dependent rise in absorbance, indicating that reducing power increased progressively with increasing sample concentration.

**Table 6:** Absorbance of standard and hydroethanolic extract of *Ipomoea batatas* leaves at various concentrations.

Concentration(*μ*g/ml)	Absorbance of standard at 700 nm	Absorbance of extract at 700 nm
10	0.215	0.164
20	0.369	0.199
30	0.604	0.366
40	0.691	0.381
50	0.826	0.471

In this study, the progressive increase in absorbance suggests enhanced electron‐donating capacity, a key characteristic of antioxidant activity. These findings align with previous investigations conducted on *I. batatas* leaves from China, where ethanolic extracts demonstrated a comparable dose‐dependent increase in absorbance in reducing power assays [4].

#### 3.5.2. DPPH Radical Scavenging Activity

**Figure 8:**
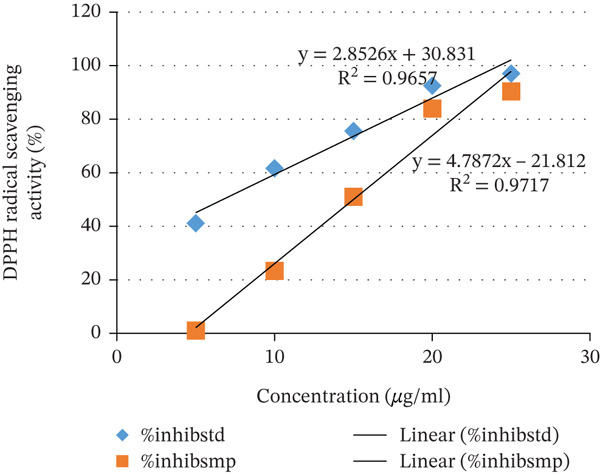
Antioxidant activity by DPPH method.

The percentage inhibition of DPPH (2, 2‐ diphenyl‐1‐ picryl‐hydrazyl) radicals by ascorbic acid (standard) and the hydroethanolic leaf extract (test sample) was determined at various concentrations (Table 7), and the corresponding graph was constructed (Figure 8).

**Table 7:** Percentage inhibition of standard and extract at various concentrations.

Concentration (*μ*g/ml)	% inhibition of ascorbic acid	% inhibition of hydroethanolic extract
5	41.19	1.06
10	61.69	23.40
15	75.59	51.06
20	92.56	84.04
25	97.07	90.42

The IC_50_ value of ascorbic acid (standard) was calculated as 6.719 ± 0.01 *μ*g/mL, whereas the hydroethanolic extract (test) exhibited an IC_50_ value of 15 ± 0.01 *μ*g/mL, demonstrating notable antioxidant potential. Because a lower IC_50_ value indicates stronger free radical scavenging activity, the standard showed greater potency compared to the extract. IC_50_ is defined as the concentration required to achieve 50% inhibition of DPPH free radicals and is commonly used to express antiradical efficiency. A smaller IC_50_ value indicates higher scavenging capacity, whereas a larger value reflects comparatively weaker activity [34].

The IC_50_ determined in the present study is lower than the value (26.76 *μ*g/mL) reported by Zhang et al. [4] for a comparable hydroethanolic extract. Conversely, research conducted on *I. batatas* leaves from Cameroon reported total antioxidant capacities ranging from 19.00 to 23.48 mg AAE/g DW, with IC_50_ values between 1.58 and 3.08 mg/mL [35], suggesting possible differences in extraction procedures or assay conditions.

In addition, a study from Indonesia [7] documented an IC_50_ value of 233.476 ± 0.01 ppm for antioxidant activity in *I. batatas* leaves, indicating relatively lower antioxidant strength. Similarly, Sultana et al. [28] reported IC_50_ values ranged from 94.6 to 115.17 *μ*g/mL (ABTS assay) and 88.83 to 147.6 *μ*g/mL (DPPH assay), further supporting the comparatively stronger antioxidant capacity observed in the present hydroethanolic extract.

### 3.6. Antimicrobial Activity

**Figure 9:**
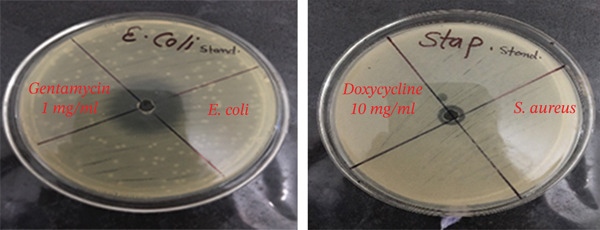
Antimicrobial activity of standard agent against test organisms (*E.coli* and *S. aureus*).

**Figure 10:**
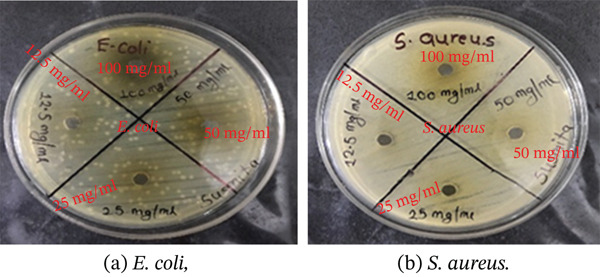
Antimicrobial activity of the plant extract against tests organisms: (a) *E. coli* and (b) *S. aureus*.

The ZOI produced by the positive control agents were evaluated against the selected test organisms (Figure 9). Gentamicin exhibited a ZOI of 30 ± 1 mm against *E. coli*, whereas doxycycline produced a ZOI of 10 ± 1 mm against *S. aureus* (Table 8). However, none of the concentrations tested of the hydroethanolic leaf extract (12.5, 25, 50, and 100 mg/mL) demonstrated any measurable zone of inhibition against either microorganism (Table 9 and Figure 10). Therefore, under the experimental conditions employed, the hydroethanolic extract of *I. batatas* (L.) Lam. leaves showed no antibacterial activity against *E. coli* or *S. aureus*.

**Table 8:** Antimicrobial activity of standard against test organisms.

S.N.	Organism	Standard	Concentration(*μ*g/ml)	Zone of inhibition(mm)
1	*E. coli*	Gentamicin	1 mg/ml	30 ± 1 mm
2	*S. aureus*	Doxycycline	10 mg/ml	10 ± 1 mm

**Table 9:** Antimicrobial activity of the plant extract against test organisms.

S.N.	Organism	Concentration of extract(mg/ml)	Zone of inhibition (mm)
1	*E.coli*	12.5	0
		25	0
		50	0
		100	0

2	*S.aureus*	12.5	0
		25	0
		50	0
		100	0

Comparable findings were reported by Pochapski et al. [8]. However, Osuntokun et al. [6] documented notable antimicrobial effects, reporting a 25 mm inhibition zone against *Salmonella typhi* at 100 mg/mL. Moderate activity (20 mm zone) was detected against *S. aureus*, *Vibrio cholerae*, and *P. aeruginosa*, whereas the least inhibition (7 mm) was recorded against *Enterococcus faecalis*. Such variations in antimicrobial outcomes may be influenced by differences in geographical origin, harvesting time, extraction techniques, and laboratory conditions.

Additionally, a study conducted in Indonesia [7] reported moderate antibacterial effects of ethanolic extracts against *S. aureus* (8.7 ± 0.01 cm) and *S. mutans* (10.2 ± 0.3 cm) at 40% concentration. Mbaeyi‐Nwaoha and Emejulu [36] evaluated peptone, aqueous, and ethanolic extracts of *I. batatas* leaves against *E. coli*, *S. typhi*, *S. aureus*, *Aspergillus niger*, *Penicillium* spp., *P. aeruginosa*, and *Klebsiella pneumoniae*. Their findings indicated that the aqueous extract exhibited comparatively greater antimicrobial effectiveness, except against *E. coli* and *Penicillium* spp., whereas ethanolic and peptone extracts demonstrated minimal inhibition, particularly against *S. typhi*.

## 4. Conclusions

Maceration of *I. batatas* leaves with 70% ethanol produced an extractive yield of 7.73%. The extract demonstrated considerable TPCs and TFCs and exhibited notable antioxidant activity, as evidenced by both the reducing power assay and the DPPH radical scavenging method.

Phytochemical evaluation confirmed the presence of biologically active secondary metabolites, which may contribute to protective effects against oxidative stress‐related conditions. Although antimicrobial activity was not observed against the tested organisms, the antioxidant findings indicate potential pharmacological relevance. Further investigation is required to isolate and characterize the responsible bioactive constituents, clarify their mechanisms of action, and evaluate their efficacy and safety through in vivo studies. Such investigations may aid in the progression of standardized plant‐derived antioxidant formulations.

## Funding

No funding was received for this manuscript.

## Conflicts of Interest

The authors declare no conflicts of interest.

## Data Availability

The data that support the findings of this study are available from the corresponding author upon reasonable request.
